# Solitary subungual neurofibroma with glomus tumor-like appearance: a case report

**DOI:** 10.1080/23320885.2020.1750018

**Published:** 2020-04-14

**Authors:** Rui Suzuki, Hiroyuki Hashimoto, Osamu Okamoto

**Affiliations:** a Department of Plastic and Reconstructive Surgery, Oita City Medical Association’s Almeida Memorial Hospital, Oita, Japan; b Department of Dermatology, Oita City Medical Association’s Almeida Memorial Hospital, Oita, Japan

**Keywords:** Solitary neurofibroma, subungual, glomus tumor-like appearance

## Abstract

We present a case of subungual neurofibroma which clinically presented a glomus tumor-like appearance. Six months after surgical resection, no clinical recurrence or pain was reported. We herein report the difficulty of its preoperative diagnosis, including literature review.

## Introduction

Neurofibroma is a benign neoplasm of the peripheral nerves, which often presents as a variant of neurofibromatosis type-1 (NF-1). The tumor arises ubiquitously in the skin of any body part; however, subungual tumors are rare. In fact, only few cases of solitary subungual neurofibroma have been reported previously. We herein report a case of solitary subungual neurofibroma in a middle-aged woman, with a preoperative diagnosis of glomus tumor.

## Case report

**Figure 1.**
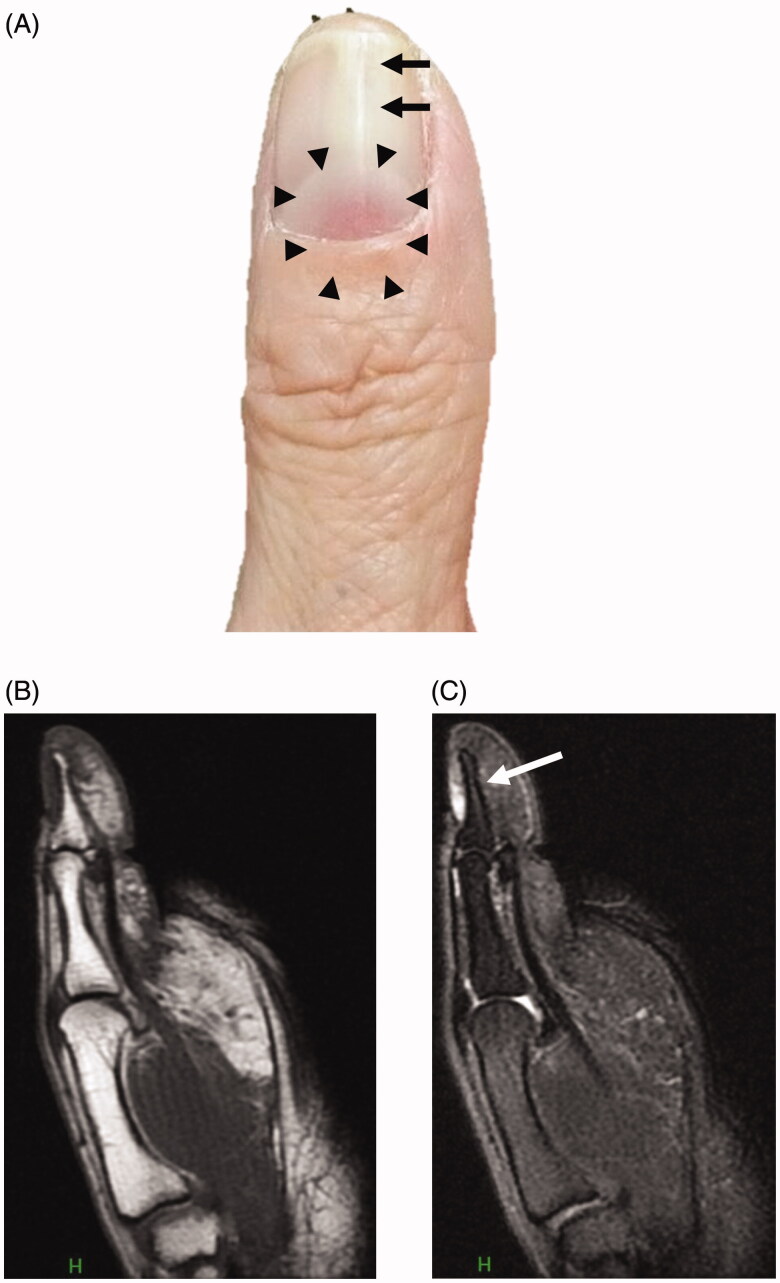
Clinical findings and MRI images. (A) A reddish mass of 4-mm diameter is seen under the nail plate (area surrounded by the triangle mark). Distal nail splitting is observed in the left thumb. (B) T1-weighed magnetic resonance image shows tumor with normal intensity. (C) T2-weighed magnetic resonance image shows high-intensity lesion. Flow void is indicated by an arrow. MRI: magnetic resonance imaging.

A 62-year-old woman presented with a two-year history of a slow-growing, painless, pale-red subungual tumor of the left thumb and distal nail splitting. She had no history of preceding trauma to the finger. The tumor was 4 mm in diameter and the nail bed proximal to the tumor was slightly elevated (Figure 1(A)). The tumor could easily be perceived. Radiographic examination showed no bone defects and magnetic resonance imaging (MRI) showed a small space-occupying lesion adjacent to the proximal part of the distal phalanx. The tumor presented with normal intensity on T1-weighed image (Figure 1(B)) and was hyperintense on T2-weighed image (Figure 1(C)). A flow void was observed in the tumor, suggesting the presence of intratumor blood flow.

**Figure 2.**
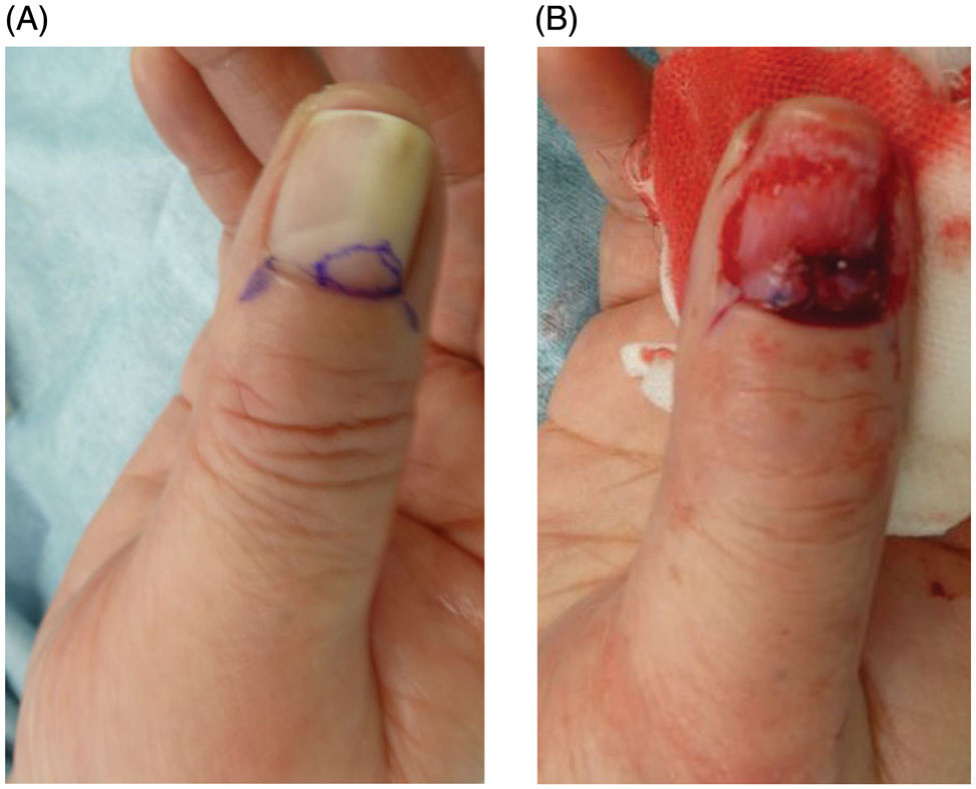
Surgical findings. (A) A reddish mass is seen under the nail plate. The excision line after the nail claw is indicated in blue. (B) After excision, skin grafting was performed from the thenar eminence.

We performed a surgical excision of this tumor under local anesthesia. After the nail claw, the skin overlying the nail matrix was lifted. On gross observation, the tumor was smooth, pale-yellow in color, well-circumscribed and did not invaded the bone. The tumor could be resected completely (Figure 2(A)). The defective nail matrix was reconstructed with a glabrous dermal graft (Figure 2(B)) taken from the thenar of the right thumb.

**Figure 3.**
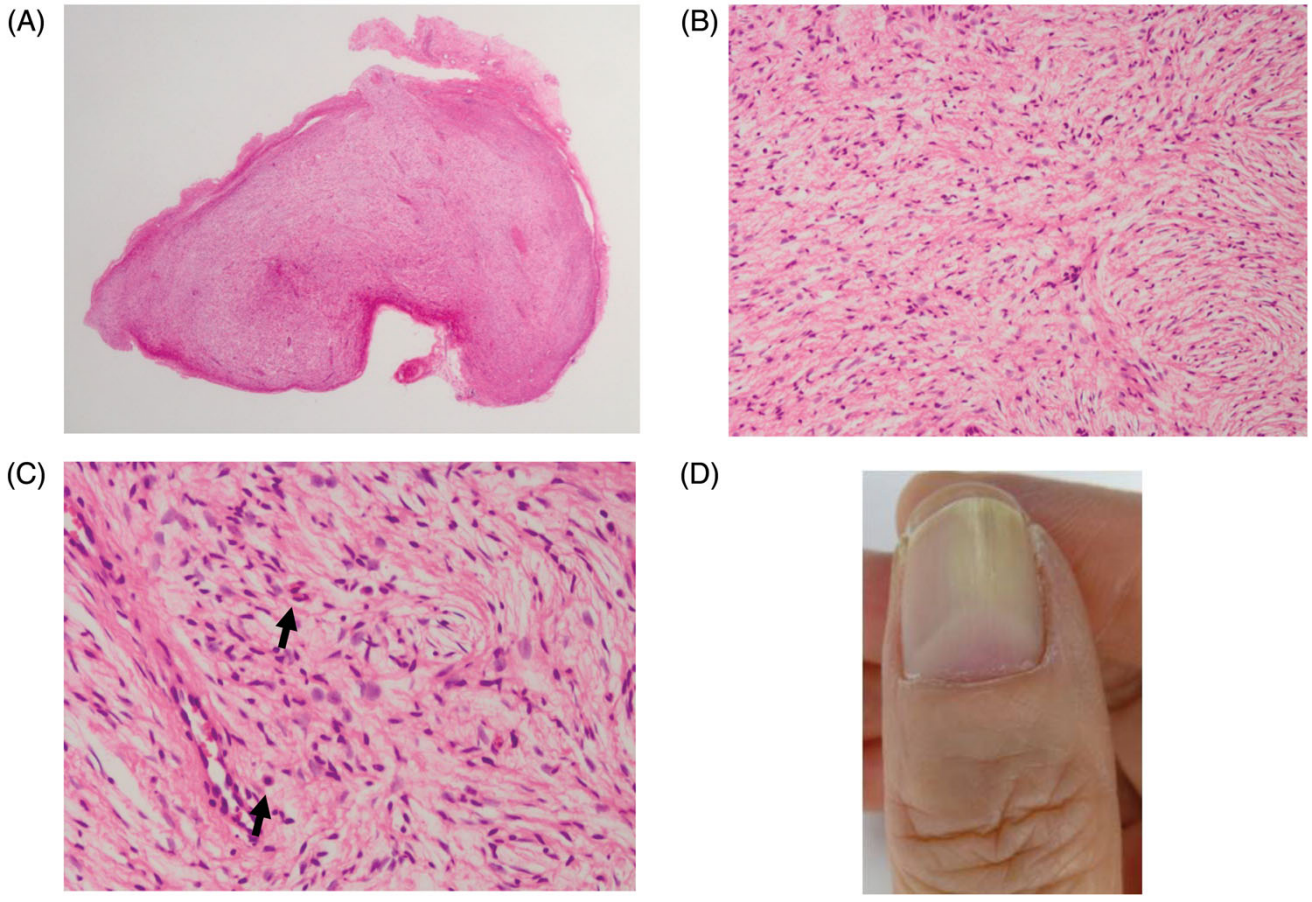
Histopathological findings. (A) A loupe image. An incompletely encapsulated tumor. (B) A middle-power view. The tumor is composed of fine collagen fibers pointed in every direction. (C) A high-power view. Nuclei of the proliferating cells are spindle- or comma-shaped. Among the tumor cells, capillaries and a small number of mast cells are dispersed (indicated by arrows). (D) Appearance of new thumb nail six months postoperatively. This observation is natural.

Histological examination of the excised specimen showed an incompletely encapsulated eosinophilic fibrous tumor (Figure 3(A)). The tumor was composed of fine collagen fibers with numerous scattered cells with spindle- and comma-shaped nuclei (Figure 3(B,C). A small number of the mast cells were observed within the tumor (Figure 3(C)). The histological diagnosis was neurofibroma and the final diagnosis of the patient was solitary subungual neurofibroma. Postoperatively, the nail re-grew with a good cosmetic result (Figure 3(D)). Tumor recurrence was not observed even at postoperative 6-month follow-up.

## Discussion

Solitary subungual neurofibroma is a rare condition. In 1981, Runne and Orfanos first described a case of neurofibroma in the nail bed [1]. Till date, only 10 cases have been reported [2,3]. Roldan-Marin et al. reported the clinical features of solitary subungual neurofibroma [4]. They described the following characteristics: (1) occurs in middle-aged females; (2) occurs in the fingers and toes; (3) asymptomatic; and (4) postoperative recurrence after surgical resection is rare. The present case had similar presentations.

A clinical diagnosis of solitary subungual neurofibroma before histological examination of the tumor is difficult because it is rare and does not usually demonstrate pathognomonic symptoms [5]. The present patient was considered to be a case of glomus tumor because of the subungual preponderance of the tumor [6]. However, because the lesion was painless, the tentative diagnosis was suspicious. Among the past reports, only two of the ten patients presented obvious pain [4,7]. Furthermore, glomus tumor causes nail plate deformity similar to that observed in the present case; therefore, it is difficult to differentially diagnose glomus tumor with subungual neurofibroma. Three previously reported cases showed thickening and elevation of the nail plate [4,8,9], while nail dystrophy was reported in five cases [3,4,7,10], including the present case. Only one case had no nail plate deformity [5]. MRI findings of normal intensity in T1-weighed image, high intensity in T2-weighed image and presence of intra-tumoral flow void were indicative of both neurofibroma and glomus tumor. Thus, differential diagnosis of these tumors by medical imaging techniques is difficult. A promising feature that supports a diagnosis of glomus tumor is the classical triad of the following symptoms: temperature sensitivity, pain and localized tenderness in the finger [6]. The absence of the pain in the present case would have completed the classic triad of glomus tumor and made diagnosis extremely reliable. We reported a case of rare solitary subungual neurofibroma with a differential diagnosis of glomus tumor. The presence or absence of the pain would be a reliable differentiator of these tumors.
